# Synthetic Antigen-Conjugated DNA Systems for Antibody Detection and Characterization

**DOI:** 10.1021/acssensors.3c00564

**Published:** 2023-07-18

**Authors:** Simona Ranallo, Sara Bracaglia, Daniela Sorrentino, Francesco Ricci

**Affiliations:** Department of Chemical Science and Technologies, University of Rome Tor Vergata, 00133, Rome, Italy

**Keywords:** DNA nanotechnology, biosensors, antibodies, DNA circuits, DNA sensors, cell-free biosensors

## Abstract

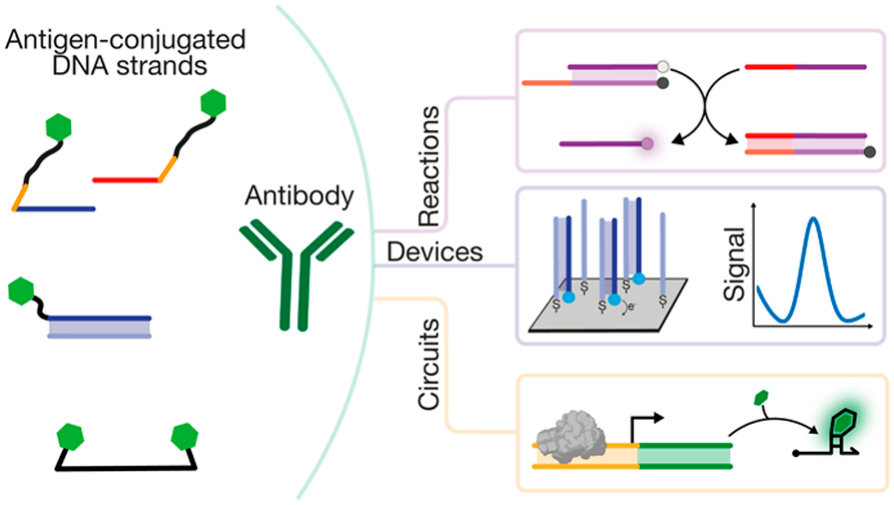

Antibodies are among the most relevant biomolecular targets for diagnostic and clinical applications. In this Perspective, we provide a critical overview of recent research efforts focused on the development and characterization of devices, switches, and reactions based on the use of synthetic antigen-conjugated DNA strands designed to be responsive to specific antibodies. These systems can find applications in sensing, drug-delivery, and antibody–antigen binding characterization. The examples described here demonstrate how the programmability and chemical versatility of synthetic nucleic acids can be used to create innovative analytical tools and target-responsive systems with promising potentials.

Antibodies are specialized proteins produced by the immune system, and specifically by white blood cells called B lymphocytes (or B cells), in response to the presence of foreign substances (i.e., antigens) such as viruses, bacteria, fungi, or parasites.^1^ The binding of an antigen to the B-cell surface induces the formation of plasma cells, that in turn secrete antibodies to attack and neutralize antigens identical to the one that triggered the immune response.^2^ Among the five immunoglobulin isotypes, immunoglobulin G (IgG) are the most abundant proteins in human serum due to their long (ca. 3 weeks) serum half-life.^3^ All IgG antibodies present a Y-shaped structure which consists of four polypeptide sequences (two heavy and two light chains) and with the antigen binding regions (associated with the light chains) separated by approximately 10–12 nm.^4^

Due to their high specificity and selectivity, antibodies are routinely used in immunoassay development for the detection of antigens.^5^ Antibodies also represent one of the most relevant classes of biomarkers for the diagnosis/prognosis of a wide range of pathologies including infectious, autoimmune, and oncological diseases.^6−8^ In addition to the importance of antibodies as disease markers, monoclonal antibodies (mAbs) are also gaining relevance as drugs in therapeutic settings.^9^ Several immuno-oncology antibodies have already been approved as drugs for the treatment of a range of tumor types including melanoma, Hodgkin’s lymphoma, and Merkel cell carcinoma.^10,11^ Bispecific antibodies (BsAb) have also been recently introduced as new promising drugs.^12,13^ These re-engineered immunoglobulins are programmed to bind two different antigens to lead to a better binding specificity and drug efficacy.^14^ Detection of such therapeutic antibodies is thus becoming increasingly important not only as a way to gain diagnostic information but also to study pharmacokinetics (PK) and toxicokinetics (TK) of immune-based therapies.

Current standard methods for the detection of antibodies, however, are based on either multistep, wash-, or reagent intensive processes (i.e., ELISA, RIA, immuno-PCR, SPR, etc.), or on qualitative or semiquantitative methods such as lateral flow immunoassays.^15−17^ The first ones are sensitive and quantitative but also require laboratory-based measurements (ELISA), hazardous reagents (RIA), and expensive instrumentation (SPR) that thus significantly limit the applicability of these techniques in point-of-care applications. Lateral flow immunoassays, on the contrary, are rapid and easy to use, but their only qualitative nature (or at best semiquantitative) limits the accessibility to quantitative information which in some cases can be relevant.

Due to the above considerations, new analytical tools that allow the rapid, inexpensive, and quantitative measurement of antibodies are urgently needed. In response, several approaches based on optical and electrochemical redouts have been recently described for antibody detection that by combining sensitivity, selectivity, and simplicity may be suitable for point-of-care applications.^18,19^ Among these, sensors based on the use of synthetic nucleic acid strands have recently emerged as a promising alternative to the currently used approaches for the detection of a wide range of molecular targets including also antibodies.^20−22^ Synthetic nucleic acids (DNA and RNA) in fact present unique advantages: they are low cost and easy to synthesize and, more importantly, their base-pair interactions are highly predictable and so it is quite straightforward to design DNA-based switches and devices that can, for example, undergo binding-induced conformational change and may be used for sensing and diagnostic applications.^23−25^ Synthetic nucleic acids are also highly versatile from a chemical point of view and they can be used as molecular scaffolds to conjugate different recognition elements (small molecules, proteins, etc.) and different signaling tags (optical or redox labels).^26^ For antibody detection, for example, synthetic DNA strands can be conveniently conjugated to the relevant antigens and with signaling tags to provide a signal only upon the binding with the target antibody. Using antigen-conjugated DNA strands can thus allow to meet the need for a sensitive, specific, and rapid approach for antibody detection. This Perspective intends to give a critical overview on the advancements made in this direction by focusing mainly on recent examples (last 5 years) of DNA-based devices, sensors, circuits, and nanostructures that employ synthetic antigen-conjugated DNA strands to respond to specific antibodies. The examples we have included in this Perspective can be divided into three major classes. Initially, we will describe systems for antibody detection that are mostly based on optical and electrochemical read-outs. In this section the majority of the examples are from the authors’ research efforts. We will then describe antibody-responsive DNA-based devices and circuits in which the binding of specific antibodies control, for example, the assembly/disassembly processes of DNA-based nanostructures or the yield of templated-reactions. Finally, we will discuss examples of antigen–DNA-based systems and structures for antibody characterization and activity control.

## Antigen-Conjugated DNA Strands for Antibody Detection

**Figure 1:**
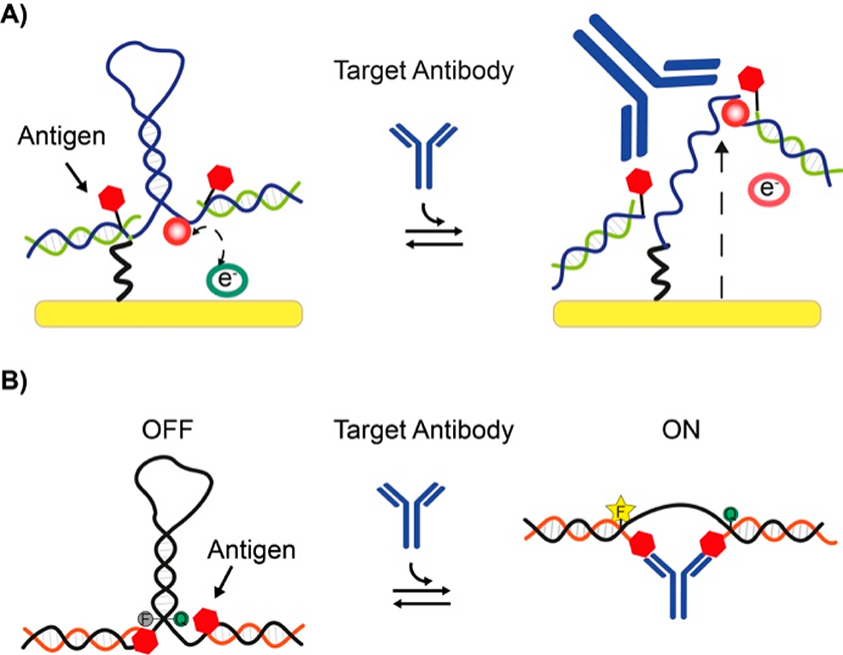
DNA-based conformational-change switches for antibody detection. These systems employ the conformational change induced by the antibody binding to two antigen-conjugated DNA strands. Such conformational switch can be linked to an electrochemical (A) or fluorescence (B) signal change. Panel A adapted with permission from ref (34), copyright 2012 American Chemical Society. Panel B adapted with permission from ref (35), copyright 2015 John Wiley and Sons.

The first examples reporting the possibility to detect antibodies using antigen-conjugated DNA strands have been demonstrated more than 10 years ago.^27−30^ In these systems (and in other follow-ups),^31−33^ the antigen-conjugated strand is attached to an electrode surface and is also labeled with an electrochemical tag. The binding of the antibody causes a change in the flexibility of the DNA probe that produces a reduction in the measured current signal. A more robust sensing mechanism was then proposed in which the antigen-conjugated DNA strand is designed to undergo a binding-induced conformational change triggered by the antibody binding to the antigen. This conformational change causes a signal change (electrochemical or optical) that can inform on the presence and concentration of the antibody. In Figure 1A is depicted the general scheme for an electrochemical antibody-induced hairpin switch in which the antigen-conjugated DNA strands are hybridized to a stem-loop hairpin DNA probe modified with an electrochemical label (usually methylene blue) and a thiol for attachment to a gold electrode.^34^ When the antibody binds to the two antigens, it will induce the opening of the stem and will force the electrochemical label away from the electrode surface. Similar sensing schemes were adapted for the detection of different antibodies also using optical signaling with the expedient of changing the electrochemical label and thiol with a fluorophore and quencher (Figure 1B).^35^ A recent addition to these sensors was reported by Merkoçi and co-workers.^36^ A Y-shaped DNA nanostructure was conjugated with antigens and redox labels and immobilized on an electrode surface. The bivalent binding of the target antibody to the antigen-conjugated strands induces a reduction in the measured faradic current.

The above-described systems, despite the slightly different sensing mechanisms, present similar advantages and disadvantages. The detection principle is direct: i.e., the sensor measures the binding of the antibody to the antigen in real-time, and equilibration is usually reached in few minutes (<10 min). Compared to other methods (such as ELISA), this is an important advantage that would make the sensors suitable for point-of-care applications (provided that the measurement can be performed with low-cost and portable instrumentation). They are specific: specificity is usually guaranteed by the antibody/antigen interaction, as no other source of signals can be envisioned in these cases. In principle, they are very versatile and could be applied to different antibody targets with the simple expedient of changing the antigen conjugated on the DNA backbone. There are, however, some drawbacks in these detection platforms that should not be overlooked. First, the detection scheme (especially for conformational change switches) requires a careful thermodynamic optimization of sensing mechanisms. This can affect the above-mentioned versatility, as changing the antigen conjugated on the DNA can alter the stability and conformational switch mechanism. In case this happens, a new optimization for each new sensor to be developed would be required. For example, the majority of these systems have been characterized with small antigens (such as small molecules and short protein epitopes). The possibility of using bigger antigens (such as proteins) would probably require a more challenging optimization of the conformational change mechanism. Another drawback is related to the direct detection scheme. While this allows very rapid response times, it is also associated with a limited sensitivity. In fact, as there are no amplification steps, the detection limit is fixed by the intrinsic instrumental limitations of electrochemical or fluorescence detection. Ironically, these are quite similar and usually do not allow to measure the antibody target below nanomolar concentrations. For some clinical applications, this concentration range is still too high especially if one has to consider the dilution of the sample often required to reduce matrix effects. For fluorescent measurements, for example, our experience is that serum samples should be diluted about 10 fold to avoid significant matrix interferences. With electrochemical detection, this dilution step is less stringent and successful experiments were also performed in whole blood.

**Figure 2:**
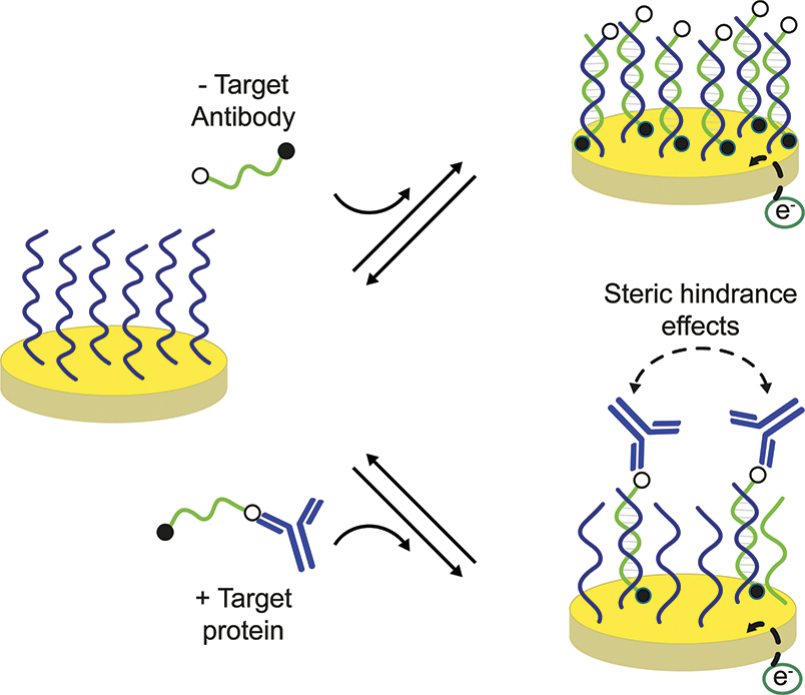
Electrochemical DNA-based steric hindrance hybridization assay for antibody detection. The electrochemical assay comprises a densely packed surface-bound capturing DNA strand (blue) and a free complementary signaling DNA strand (green) that is dually labeled with a small recognition element and a signaling redox label. The binding of the target antibody to the antigen-conjugated strand produces a steric hindrance effect that reduces the signaling DNA strand binding efficiency to the capturing strand, resulting in a decrease of the faradic current. Adapted with permission from ref (37), copyright 2015 American Chemical Society under open access license.

In addition to the above approaches, other sensing principles have been designed utilizing antigen-conjugated DNA strands. One example in this direction employs the effect of the antibody steric hindrance on the hybridization efficiency between two DNA strands.^37^ More specifically, an antigen-conjugated nucleic acid strand is designed to hybridize to a redox-labeled complementary DNA probe immobilized on a gold electrode. When the antigen-conjugated strand is bound to an antibody, its hybridization efficiency (the kinetics of the binding) is reduced and this can be conveniently measured electrochemically (Figure 2). This system joins a homogeneous reaction step between antibody and antigen with a heterogeneous signaling event (electrochemical) which can lead to advantages in terms of noise reduction and use in complex media. The sensitivity limitation discussed above for other systems remains the same, even if recently the use of nanostructured electrodes has been reported to provide lower detection limits (picomolar concentration range) thanks to the larger surface areas of the electrodes.^38^

**Figure 3:**
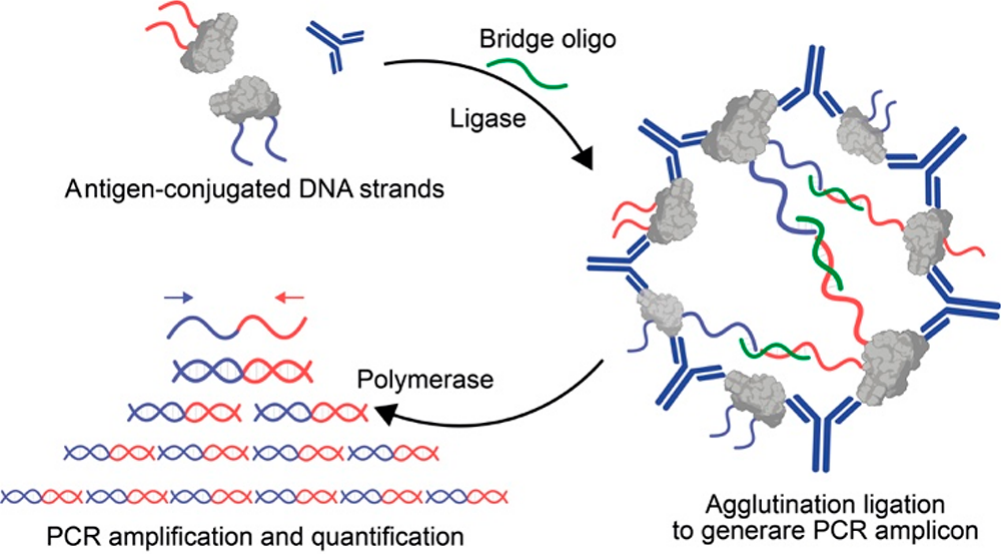
General scheme of antibody detection by agglutination-PCR (ADAP) in which the antibody binding to the antigen-conjugated DNA strands triggers the formation of a duplex DNA reporter that can be amplified and quantified by PCR. Adapted with permission from ref (39), copyright 2016 American Chemical Society under open access license.

An approach that could solve the sensitivity limitation has been proposed by the group of Bertozzi and is inspired by the proximity ligation assay.^39^ The approach is named antibody detection by agglutination-PCR (ADAP) and uses antigen-conjugated DNA strands that, in the presence of the target antibody, aggregate to generate a duplex DNA reporter that can be amplified and quantified by PCR (Figure 3). Compared to the previously described examples here, the signal amplification step due to PCR leads to a much better sensitivity (detection limits in the low pM range). The versatility of the system is demonstrated using antigens of different size (from 0.24 kDa to 150 kDa) with similar results in terms of sensitivity. Moreover, it is also possible to multiplex the assay by designing orthogonal PCR probes. However, the high sensitivity is achieved at the cost of a more time-consuming assay (about 3 h) and requires bench instrumentation such as a PCR thermocycler.

**Figure 4:**
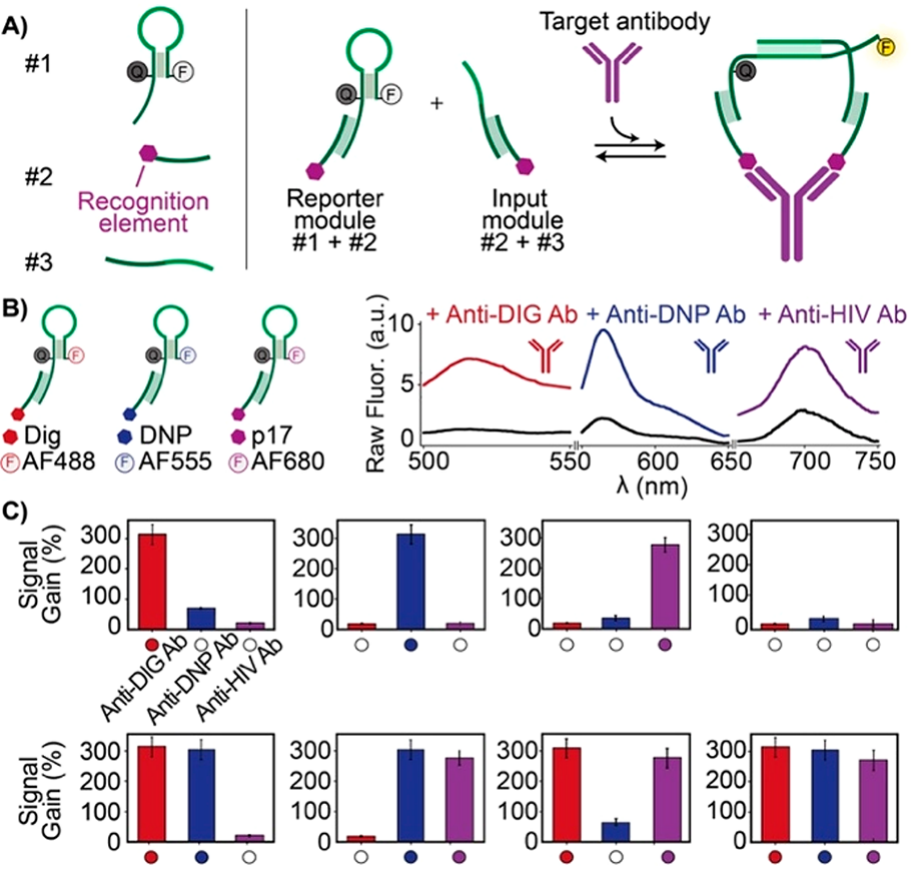
Fluorescence-based nucleic acid platform for antibody detection. (A) The platform consists of two nucleic acid modules (reporter and input module) that are programmed to colocalize in the presence of the target antibody, providing an increase in the fluorescence signal. (B) The modular nature of the platform allows the detection of different antibodies (anti-Dig, anti-DNP, and anti-HIV antibodies) by simply changing the recognition element conjugated to the DNA strands and using reporter modules with different fluorophore/quencher pairs. (C) Signal gain of the antibody-responsive modules obtained by challenging various combination of the three target antibodies. Adapted with permission from ref (40), copyright 2018 American Chemical Society.

Following these demonstrations of antigen-conjugated DNA strands for antibody detection, new reports have proposed additional sensing strategies with the objective to achieve better sensitivity and versatility. Recently, for example, our group has reported a system that couples the advantageous features of DNA-based conformational switches with those of colocalization based methods.^40^ Specifically, the system consists of two modules. The first module (reporter module) is formed by the hybridization of a synthetic fluorophore/quencher labeled stem-loop DNA switch (strand #1, Figure 4A) and a synthetic DNA strand conjugated with a recognition element (i.e., antigen) (strand #2, Figure 4A). The second module (input module) is a ssDNA sequence complementary to the loop portion of strand#1 and conjugated to another copy of the same recognition element (strand #3, Figure 4A). In the absence of the target antibody, the two modules are designed so that strands #1 and #3 have a poor binding affinity, and so strand #1 remains in its stem-loop conformation, providing a low fluorescence signal. The binding of the target antibody to the antigen-conjugated strands induces the colocalization of the reporter and input modules, increasing their local concentrations and triggering their hybridization (Figure 4A). This results in a conformational change that opens the stem-loop conformation and leads to an increase in the fluorescence signal. The system employs the concept of colocalization, that has been vastly employed in sensing applications^41^ and is particularly suitable in this context due to the bivalent nature of antibodies. The advantages of this system are similar to the previously reported sensors. It is rapid (response time is approximately 5 min) and specific (no significant cross-reactivity) and can be applied to the detection of different antibodies. Moreover, by using different fluorophore/quencher pairs, the multiplexed detection of different target antibodies in the same solution can be achieved (Figure 4B,C). Using a 1:10 dilution, the system also works well in blood serum and blood plasma. As a demonstration of this, the detection of trastuzumab (a growth-inhibitory humanized monoclonal anti-HER2/neu) in patients treated with this drug has been demonstrated.^42^

Despite the above advantages, similar drawbacks as those described for other direct antigen-conjugated DNA assays can be found. First, there is no amplification step so sensitivity remains in the nanomolar range. This, coupled with the need of a dilution step (1:10) to avoid a high fluorescent background from the sample, means that the sensor cannot be employed when antibody concentration is expected to be in the pM range. Also, it has to be demonstrated the possibility of using whole proteins as recognition elements and this is key to the actual versatility of these systems. Finally, the colocalization approach at first sight can present an intrinsic limitation: that is, only a portion of the total antibodies available in solution will actually give a signal, as each antibody can, in principle, bind indistinctly the two antigen-conjugated strands, thus resulting in the formation of nonsignaling complexes (by binding the same antigen-conjugated strand). However, in our experience, this is often not a major problem. In fact, the binding by one antibody to the two different antigen-conjugated strands is thermodynamically favored, as this binding is associated with the hybridization event. This makes the contribution of nonsignaling antibody/antigen binding events to the overall signal negligible.

Trying to improve sensitivity and especially to avoid dilution steps that could affect the final detection limit, a signal-ON electrochemical sensor for antibody detection has been recently developed using a similar colocalization principle.^43^ In this example an antigen-conjugated strand (capture strand) is anchored to a gold disposable electrode while a DNA strand conjugated at the two ends respectively with a redox tag and an antigen molecule (output strand) is free in solution. The target antibody colocalizes the capture and the output strands, leading to the formation of the duplex complex and thus bringing the redox label in close proximity to the electrode surface. This results in an increase in the measurable electrochemical signal as a function of the target antibody concentration. The system is especially interesting, as electrochemical detection can be far more convenient than fluorescence due to the portable instrumentation, the ease of operation, and the lower level of possible interferents.

In the absence of a chemical or enzymatic amplification step, improved sensitivity can be only achieved with more sophisticated instrumentation. A demonstration in this context has been given by the group of Tinnefeld that reported a DNA origami-based sensor for antibody detection.^44^ Specifically, the system comprises a DNA origami nanoantenna that incorporates the previously described optical antibody-responsive switch^35^ and provides a fluorescence signal enhancement. Compared to other methods of creating plasmonic fluorescence enhancement, the use of synthetic DNA offers the possibility to rationally program the placements of nanoparticle and the antibody-responsive switch in a programmable way. The platform allows to decrease the limit of detection of the nanoswitch to the picomolar range and can, in principle, be adapted for multiplexing detection.

Another approach for the detection of antibodies using antigen-conjugated DNA strands has been demonstrated by Rant and co-workers. The platform (named “switchSENSE”) is able to analyze protein size and conformation by measuring the instantaneous velocity of an antigen-conjugated DNA strand that is electrically actuated to oscillate at high frequencies on a chip.^45^ The binding of a target protein to the nanolever adds an additional friction that slows the nanolever movement and in turn provides a measurable signal change. Based on this principle, a DNA-based surface biosensor with integrated microfluidic channels to analyze the binding kinetics of therapeutic antibodies to TNF-α cytokine has been recently reported.^46^ The DNA-based surface biosensor is composed of dynamic DNA nanolevers bound to microelectrodes at one end and presenting TNF-α molecules at the other end. The switching between lying and standing orientation of the nanolevers has been tuned by applying AC potentials to the microelectrode to achieve a potential-dependent regulation of molecular motion. The binding of therapeutic antibodies to TNF-α induces a decrease of nanolever switching speed (increase of the hydrodynamic friction) that can be conveniently measured, providing a way to quantify the target antibody. This approach can be, in principle, easily extended to other antibodies for which the specific recognition binding event induces a variation of the hydrodynamic friction of a DNA nanolever. The switchSENSE platform has the advantage of being already commercialized and fully operational. However, it might not be the best approach for a point-of-care system, as the instrument is not portable and cannot be considered low cost. The system thus appears as a very good alternative for characterization of antibodies especially in the immunotherapy drug industry. It should also be pointed out that for this approach the same sensitivity limitations apply: that is, the system measures a direct binding of the antibody to an antigen conjugated to a DNA and so no amplification steps are present that would allow to reach pM/fM detection limits.

**Figure 5:**
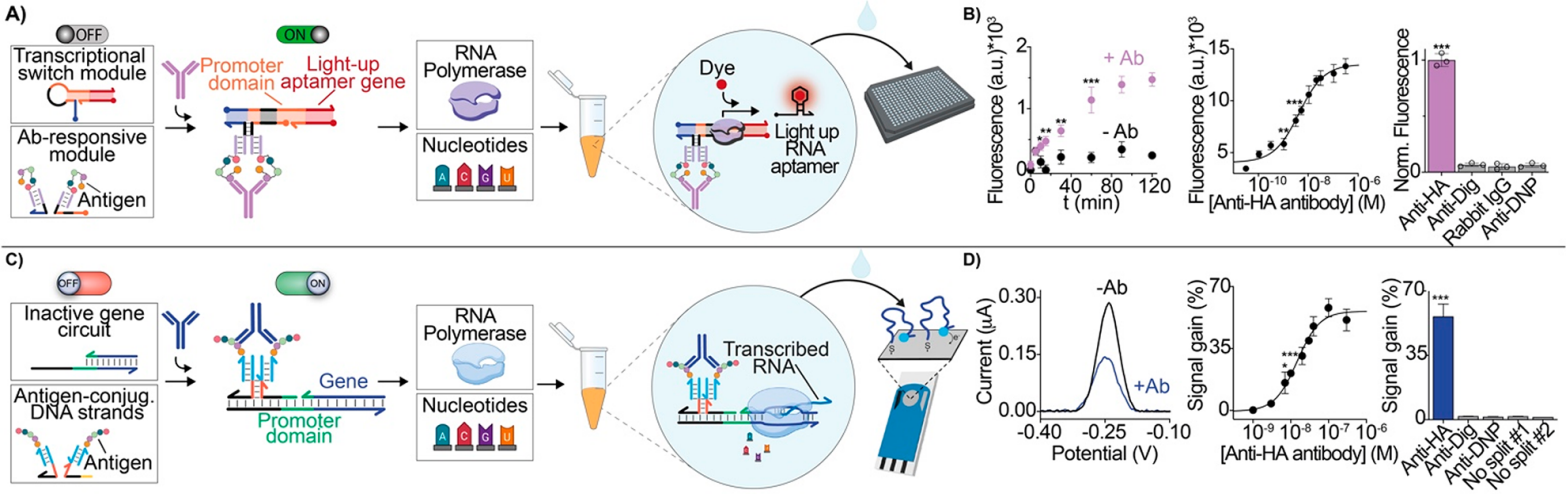
Cell-free biosensors for antibody detection. (A) The optical cell-free biosensor comprises a transcriptional module and an antibody-responsive module. The antibody-activated transcriptional switch can transcribe, in the presence of RNA polymerase and nucleotides, a reporter light-up RNA aptamer that signals the presence of the target antibody. (B) Anti-HA antibody detection employing as recognition element a short peptide (9-residue) present on the surface of the influenza virus. (C) Electrochemical cell-free biosensors. The antibody-responsive gene is activated only in the presence of the specific antibody that, by binding the two antigen-conjugated DNA input strands, reconstitutes the T7-RNAP promoter region. The transcribed RNA output strand can be detected using a disposable electrode on which a redox-labeled DNA probe is immobilized. (D) Electrochemical detection of anti-HA antibody in complex matrix sample. Panels A and B adapted from ref (53), copyright 2022 American Chemical Society under open access license. Panels C and D adapted from ref (54), copyright 2023 John Wiley and Sons.

Recently, cell-free transcription/translation biosensors have emerged as innovative analytical devices.^47^ These systems are based on synthetic genes that can be activated in the presence of a specific target and trigger the in vitro transcription of a signaling RNA strand or the translation of a signaling protein. A wide range of cell-free biosensors for the detection of specific nucleic acid sequences,^48−50^ small molecules,^51^ and metal ions^52^ have been reported, demonstrating the potentialities of these systems as novel analytical devices. We have recently reported the first examples of cell-free transcription sensors for antibody detection.^53,54^ In a first report we have designed a programmable antigen-conjugated DNA transcriptional switch that induces the cell-free in vitro transcription of a light-up RNA aptamer in the presence of a specific target antibody.^53^ The system comprises two modules: the transcriptional switch module and the antibody-responsive module (Figure 5A). The transcriptional switch module consists of a DNA-based duplex designed to contain the double-stranded portion transcribing for a light-up RNA aptamer, the T7 RNA polymerase (T7-RNAP) promoter domain, and an additional switching domain encoded in the hairpin structure. Part of the T7 promoter sequence is hidden into the hairpin structures so that in this conformation the T7 promoter domain is not accessible to the T7-RNAP enzyme and thus transcription is prevented. The antibody-responsive module instead comprises a pair of antigen-conjugated strands designed to colocalize and form a bimolecular complex exclusively upon the binding of a specific target antibody. This complex leads to a conformational change of the transcriptional switch through a toehold-mediated strand displacement reaction that results in the reconstitution of the complete promoter domain. This ultimately triggers the transcription of the RNA light-up aptamer, leading to a fluorescence signal that informs on the presence and concentration of the target antibody (Figure 5B).

In a second example, we have developed an electrochemical cell-free biosensor based on an antigen-conjugated synthetic gene.^54^ Specifically, the responsive synthetic gene is designed to contain an incomplete T7 promoter region that prevents efficient transcription by the T7-RNAP. The promoter region can be reconstituted only upon the binding of the antibody to two antigen-conjugated DNA input strands. The RNA output strand transcribed in the presence of the target antibody hybridizes to a redox-modified probe strand attached to a disposable electrode. This, in turn, generates a change in the measured electrochemical signal (Figure 5C). Using this system, we have detected three different antibodies (including the influenza-relevant anti-HA antibody) directly in complex matrix samples (Figure 5D). The two above examples based on cell-free transcription would in principle present the important advantage of coupling the antibody/antigen binding event with an enzymatic reaction (i.e., DNA to RNA transcription). This could allow better sensitivities in comparison with the approaches in which the antibody/antigen binding is measured directly without any amplification step. Unfortunately, however, we have to note that this was not the case. In fact, both the fluorescent and electrochemical cell-free transcription systems show sensitivities in the nanomolar range that are comparable with those of other direct sensing approaches. The reason for this is not totally clear. It could be that the amplification associated with the enzymatic transcription reaction is somehow nullified by the more complex sensing scheme that requires not only a colocalization event but also the reconstitution of an active promoter domain.

## Antibody-Responsive DNA-Based Devices, Circuits, and Structures

**Figure 6:**
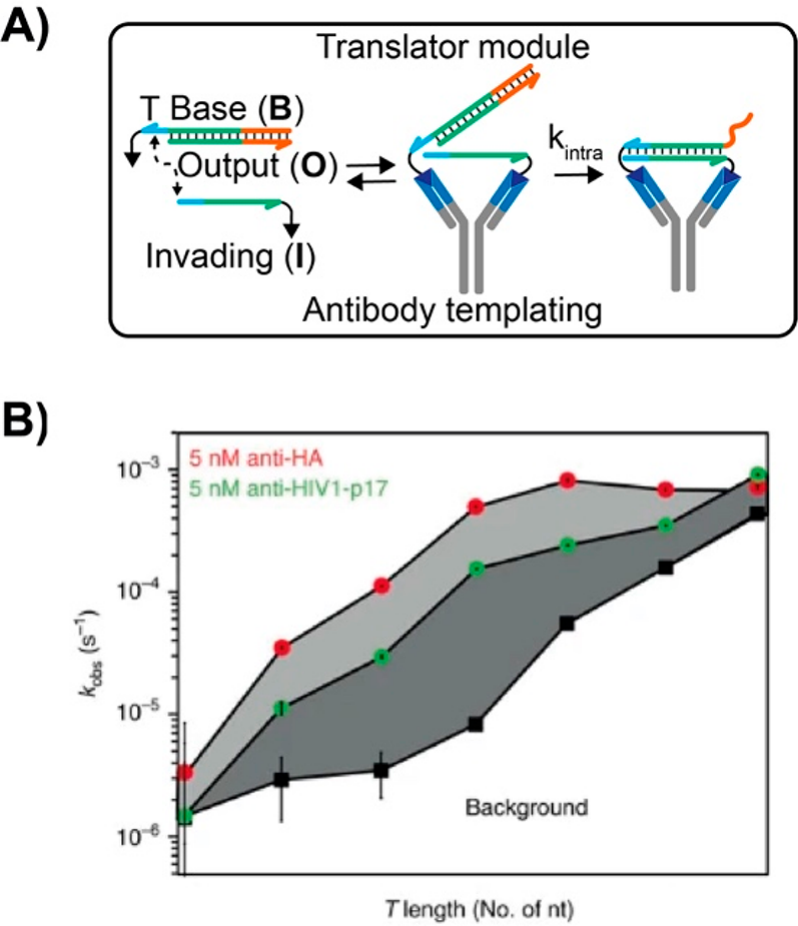
Antibody-templated strand exchange reaction. (A) The binding of the antibody to antigens conjugated to the duplex complex (B) and invading strand (I) induces the release of a DNA output strand, thus triggering the downstream toehold-mediated strand displacement reaction. (B) Apparent first-order constant (*k*_obs_) for two different antibodies obtained by using different toehold length portions. Adapted from ref (56), copyright 2017 John Wiley and Sons under open access license.

The unique programmability of synthetic nucleic acid strands can also be conveniently used to rationally engineer DNA-based circuits programmed to respond to multiple inputs and, in turn, provide optical or electrochemical readouts or activate a downstream reaction.^55^ One clever example of such possibility is represented by a DNA-based circuit controlled by specific antibodies that has been recently reported by the group of Merkx.^56^ The DNA circuit translates the presence of an antibody into a single-stranded DNA output through a DNA strand exchange reaction. The circuit involves the use of a prehybridized duplex complex and of an invading strand each conjugated to an antigen. The binding of the antibody to the two antigens triggers a strand displacement reaction between the invading strand and the duplex complex, thus blocking the antibody binding sites and promoting the release of a DNA output strand (Figure 6A). A detailed characterization of the system as a function of toehold portion length, antibody–antigen affinity, and concentration allows to establish a model describing the thermodynamics and the kinetics of the reaction (Figure 6B). The authors have also demonstrated the multiplex detection of anti-HA and anti-HIV1-p17 antibodies based on a set of Boolean logic operators, thus proving the system particularly suitable for antibody-based diagnostics.

The system couples a colocalization mechanism to DNA-based reactions and provides the first demonstration of an antibody-induced strand exchange reaction. From an analytical point of view, it should be noted that the same limitations as those described for other systems are present (i.e., no signal amplification and need to demonstrate applicability with larger recognition elements).

**Figure 7:**
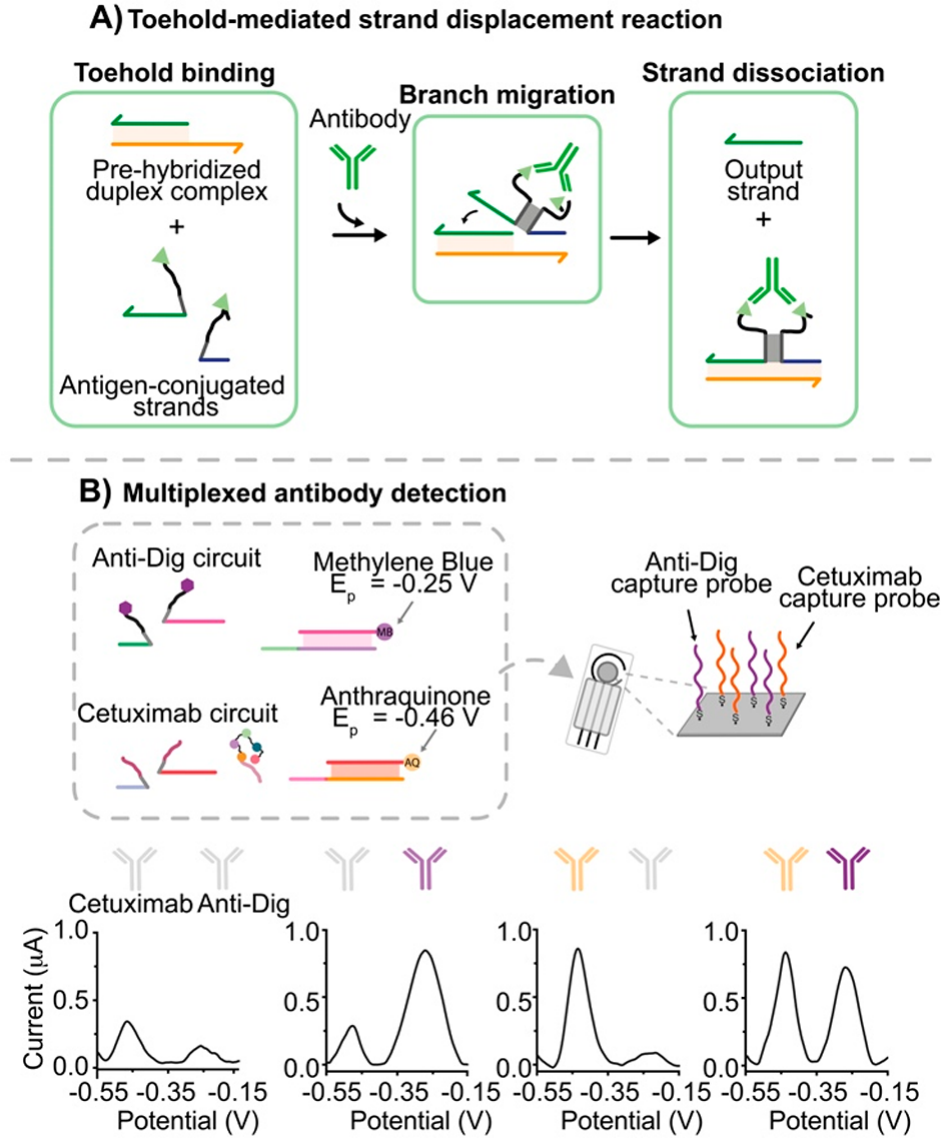
Antibody-controlled toehold-mediated strand displacement reaction. (A) General mechanism of antibody-responsive strand displacement reaction. (B) Electrochemical platform for the multiplexed detection of anti-Dig (orange) and cetuximab (purple) antibodies comprising two orthogonal-responsive circuits in which the released output strands are labeled with two noninterfering redox labels. Panel B adapted from ref (58), copyright 2021 American Chemical Society under open access license.

Following this work, our research group has also demonstrated an antibody-responsive DNA-based circuit.^57^ To do so, we have redesigned the DNA invading input strand of a classic strand displacement reaction by splitting it into two separated strands. The two split strands contain (i) a complementary stem-forming portion, (ii) the toehold and invading domains respectively, and (iii) an antigen for the target antibody conjugated at one end. The bivalent binding of the antibody to two antigen-conjugated split strands induces their colocalization and the reconstitution of the functional unit (invading strand) able to initiate a strand displacement reaction. (Figure 7A). Orthogonal DNA-based reactions can be designed that can be regulated by different antibodies independently in the same solution without crosstalk.

An advantageous feature about these circuits is that they are quite versatile in terms of signaling. In fact, the output strand can be labeled with different signaling tags and give different signals upon antibody binding. For example, recently we have adapted this DNA circuit to an electrochemical platform to enable the electrochemical quantification of multiple antibodies (Figure 7B).^58^ It should be noted that the analytical features of these systems in terms of sensitivity and specificity do not differ much from those of previous examples based on different mechanisms. Despite this, we are particularly keen of this approach because it is virtually leakless (i.e., the background signal is very low), as the output can only be generated when the antibody colocalizes the two split input strands. This is not always true for other colocalization-based approaches and for methods based on conformational-change mechanisms that often display background signals that affect the overall sensitivity of the system. Another advantage of such antibody-induced reactions is only marginally related to sensing. In fact, DNA strand displacement reactions are commonly employed in the field of DNA nanotechnology to control many different processes such as the assembly of DNA-based structures or the operation of DNA-based devices. Similar antibody-responsive DNA-based circuits can thus be linked to other processes, making them responsive to the presence of a specific antibody. To demonstrate this, we have designed a system in which the output strand of the strand displacement reaction can trigger the assembly or disassembly of DNA-based nanostructures (i.e., DNA nanotubes). The approach is highly versatile and allows to control the orthogonal assembly and disassembly of different structures with multiple antibodies in the same solution.^57^

**Figure 8:**
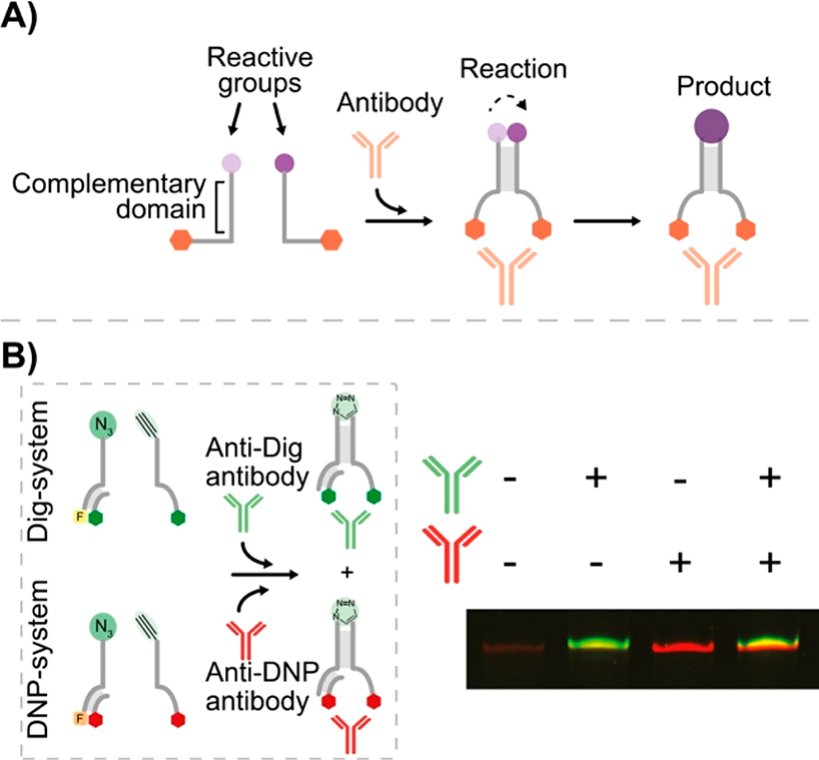
Antibody-controlled DNA-templated chemical reaction. (A) Complementary DNA templating strands designed to hybridize only in the presence of a specific antibody, thus leading to a chemical reaction. (B) Using two DNA circuits responsive to anti-Dig and anti-DNP antibodies respectively, the synthesis of two different products has been achieved in an orthogonal way. Adapted from ref (59), copyright 2020 Springer Nature under open access license.

In another recent example, our research group has reported the possibility to control chemical reactions using IgG antibodies as a cotemplating agent.^59^ To do that, we used two antigen-conjugated DNA strands modified at the other end with two reactive groups. The bivalent binding of the antibody to the antigen-conjugated strands promotes their hybridization, thus ultimately triggering the chemical reaction (Figure 8A). We initially triggered the classic biorthogonal chemical reaction copper(I)-catalyzed azide–alkyne cycloaddition (CuAAC) by designing an antibody-templated strand that employs the small molecule hapten digoxigenin (Dig) as recognition element and using the specific anti-Dig antibody. The versatility of our approach leads not only to trigger a second reaction (phosphoramidate ligation reaction) using a different recognition element/antibody couple but also to achieve an orthogonal control of two different templated reactions in the same solution with two different antibodies (Figure 8B). More recently, in a follow-up work in collaboration with the Gothelf group, we have extended the same approach to translate protein–protein binding events into DNA-templated reactions.^60^ Antibody-controlled templated reactions can be also useful for sensing applications in case the reaction leads to a measurable output (for example optical), but many other possible applications can be envisioned. For example, it would be possible to design templated reactions that lead to the formation of an active compound only once a target antibody binds to the two templating strands. This could have potential applications in the pharmaceutical industry and could be used for biomarker-induced production of drugs. A limitation in this direction could be, again, the stoichiometric ratio between the antibody and the templating strands that might give too low amount of the produced compound.

**Figure 9:**
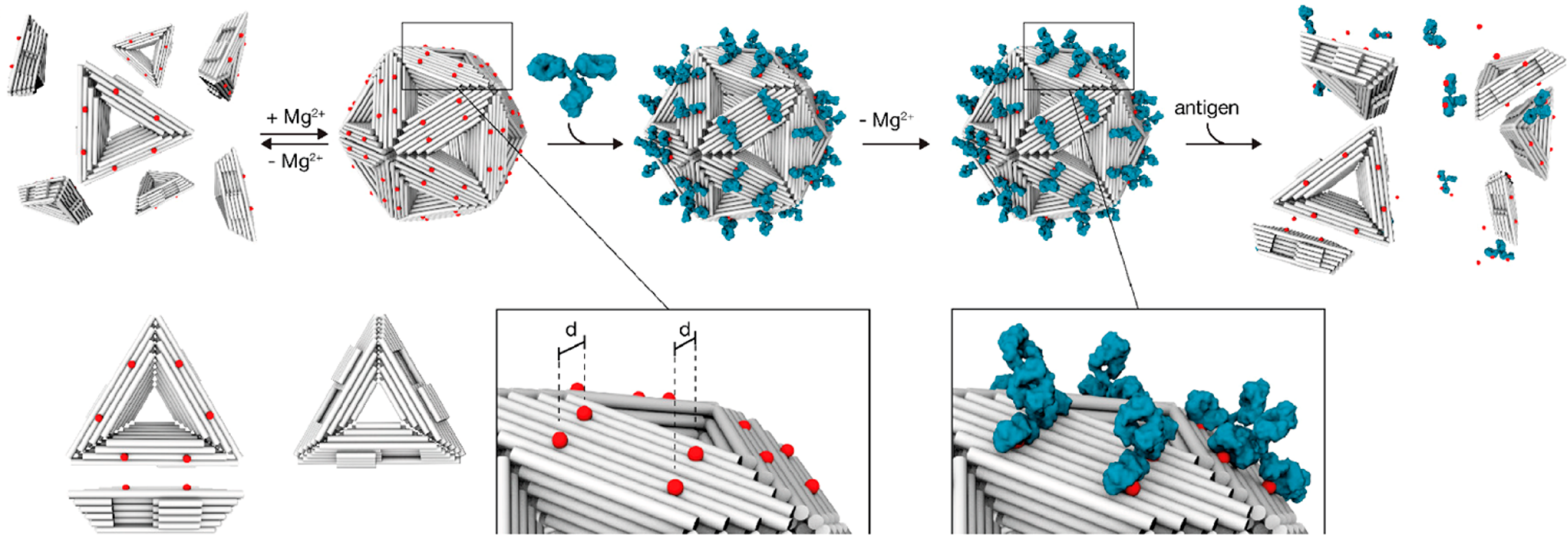
IgG antibody-mediated reconfiguration of icosahedral DNA origami shells. General scheme of antigen-decorated DNA origami shell switching mechanism upon antibody bivalent binding/unbinding process to antigen pairs. Double-helices are indicated as cylinders, antigens as red circles, and IgG antibodies as blue y-shaped objects. In the inset, “d” denotes the pairwise antigen spacing (ca. 10–12 nm). Adapted from ref (61), copyright 2021 American Chemical Society under open access license.

New avenues for diagnostic and therapy could be offered by the possibility to program functionality of DNA-based devices, such as the release of a molecular cargo, in response to the antigen/antibody interaction. Dietz and co-workers have recently reported a clever example in which the reconfiguration of antigen-decorated DNA-based nanostructures can be controlled by IgG antibodies.^61^ The DNA-based nanostructure is an icosahedral DNA origami shell of 20 identical DNA origami triangle subunits decorated with antigens at a distance compatible with the separation of the two binding sites of the IgG antibody (ca. 10–12 nm).^4^ Under certain conditions (i.e., variation of Mg^2+^ concentration), the DNA nanostructure is forced to switch to a conformation where the antigen’s distance is no longer compatible with the space between the binding sites of the IgG antibody but the shell does not disassemble unless the antibodies dissociate (Figure 9). A concentration-dependent antigen-triggered disassembly of DNA origami shells for two different antigen/antibody couples (Dig/anti-Dig antibody and DNP/anti-DNP antibody) and an AND logic-gated actuation of DNA origami by using a combination of antigens has been achieved. Furthermore, a possible application of this strategy for drug release has been demonstrated by programming the shells in order to break and release a viral cargo in response to the presence of specific antigens. The mechanism is robust and, in principle, adaptable to other higher-order assemblies in which the simple antigen-spacing criteria is satisfied. For example, DNA-based nanostructures of different shapes can be programmed to burst (disassemble) not only in response to free antigens but, in principle, also upon recognizing certain cell surface markers to achieve cell-targeting and controlled drug release. Nevertheless, the DNA origami technology still remains limited in terms of practical usability due to the high cost of production and limited stability of the structures.^62^ As these issues are being currently investigated by many research groups, we are confident that a solution can be found and DNA origami can find different applications in real settings.

## DNA-Based Systems for Antibody Activity Control and Characterization

Antigen-conjugated DNA systems can be applied not only for sensing but also to directly control antibody activity. Merkx and co-workers have demonstrated the design of bivalent antigen-conjugated DNA strands (named “DNA-locks”), in which the two antigens (here peptides) span a distance of 10–12 nm and can be used to reversibly control antibody activity.^63,64^ The DNA locks are dsDNA designed to provide an efficient bridge between the two antigens, thus leading to a stable interaction between antibody and ligand. The binding of the DNA lock to the antibody makes the antibody binding sites unaccessible to other antigens. This blockage can be reversed by using protease-cleavable antigen peptides^64^ or by introducing a toehold portion sequence to achieve a strand displacement-mediated restoration of the antibody activity.^65,66^

To expand the range of inputs to control antibody activity, the use of external triggers such as light and pH has been recently demonstrated.^67,68^ In a first example, a photoresponsive moiety has been introduced in the DNA lock to achieve an antibody activity regulation triggered by light. In this case, the peptide–DNA lock consists of 20 bp dsDNA conjugated with hemagglutinin (HA) epitope to the 5′-ends of both strands and the photolabile 3-amino-3-(2-nitrophenyl)propionic acid peptide. Irradiation of the antibody–DNA lock complex at 365 nm for 10 min induces the cleavage of linker between the peptide epitope and the DNA strand and almost completely restores the antibody activity to target cell-surface receptors. In another example, the introduction of programmable pH-responsive DNA triple helix structures (that involve both Watson–Crick and Hoogsteen interactions) in the linker sequence of the DNA locks allows to control the antibody activity either by an increase or a decrease of the solution’s pH.

The above-described systems represent compelling examples on how antigen-conjugated DNA strands can be used not just for sensing applications. A limitation that still needs to be addressed is how similar systems can be delivered into a human body and still preserve the capacity to control antibody function. While many different methods can be used for delivery and stabilization of DNA strands it will be not an easy task to control the location and the amount needed for antibody locking. A step forward in this direction would also be to genetically encode antigen-conjugated DNA strands.

Synthetic DNA-based devices can also be used to characterize antibody binding activity. As a demonstration of this, Högberg and co-workers have recently introduced a new method to characterize antibody activity by exploring the relation between the structural flexibility and the ability of an antibody to bind its antigens. Such dynamic interplay has been investigated using DNA origami structures patterned with antigen molecules (i.e., digoxigenin) immobilized on a surface plasmon resonance (SPR) chip and studying the interaction with the IgG anti-Dig antibody.^69^ Different distances between the two antigens (from 3 to 17 nm) have been investigated, showing a peak of higher binding affinity at 16 nm. The platform also allows to determine how other factors, apart from antigen spatial distribution, can affect the antibody binding affinity. For example, differences in the constant region of the antibody provide a variable flexibility that greatly influences the binding strength to patterned DNA origami. Based on the knowledge of antigen patterning, type of antibody involved, and the antigen/antibody affinity, this method allows to predict how an antibody will target a wide range of antigens on different nanoscale densities, such as on cell receptors or pathogens.

**Figure 10:**
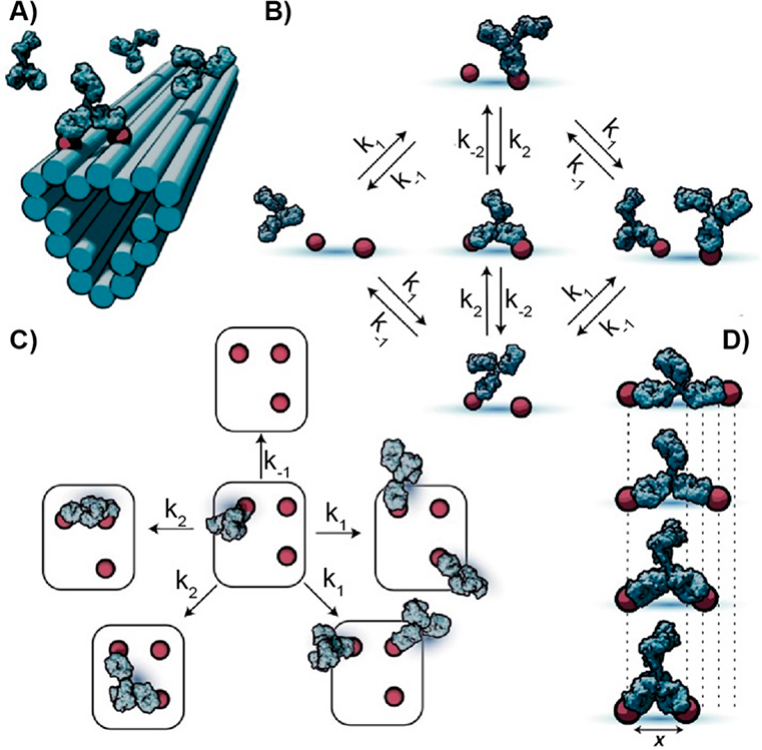
Antigen-conjugated DNA origami for antibody/antigen binding characterization. (A) Representation of antigen-conjugated DNA origami nanostructures. (B) Monovalent and bivalent biding state described by the Markov model. (C) Model extension that separates the system into elementary transition states as different combinations of empty and monovalently or bivalently occupied antigens. (D) Pairs of antigens separated by different lengths that affect antibody-binding kinetics on the chance of bivalent interconversion. Reprinted by permission from ref (70), copyright 2022 Springer Nature under open access license.

More recently, the same group has also developed a mechanistic model that describes the antibody interactions with patterned antigen substrates (Figure 10A).^70^ The collected SPR data have been converted into a flexible model that considers the antibody binding to the antigens as a discrete Markov process comprising two distinct states: monovalent and bivalent antibody–antigen complexes (Figure 10B). This model describes the transitions between these states as governed by elementary rates to simplify the comprehension of the complex interactions of patterned surfaces with biomolecules containing multiple binding domains (Figure 10C). The model foresees that gradient (or geometry) in antigen spacing on the patterned DNA origami nanostructures can guide antibody movement in the direction of more stable spacing (Figure 10D). The stochastic-predicted walking mechanism and the molecular programmability of nucleic acid systems could be of utility for the rational design of molecular machines and vaccines.

Characterization of the binding of antibody-functionalized DNA nanostructures to different receptors has been also demonstrated by De Greef and co-workers.^71^ The authors have focused on clinically relevant receptors including the programmed cell death protein 1, the epidermal growth factor receptor, and the human epidermal growth factor receptor 2 and have studied the effect of an incorporated protein ligand onto a DNA nanostructure on the affinity for the receptor. A systematic characterization of a DNA nanostructure decorated with fluorescently labeled antibodies confirms the absence of effect toward the native binding affinity of the antibody for its receptor and highlights the influence of nanostructure size and DNA handle location. By monitoring the DNA nanostructure–cell interactions, at increasing DNA nanostructure size a lower receptor binding efficiency has been found as a consequence of steric hindrance effect. Indeed, cell surface composition and density act as a natural barrier that influences receptor accessibility. This work provides insights on the parameters to design programmable DNA origami nanostructures in which the receptor accessibility can be modulated in a controlled fashion for optimal cellular targeting. Characterization of antibody binding using antigen-decorated DNA structures in our opinion represents a very interesting niche application with different advantages. Thanks to the programmability of DNA/DNA interactions, it is possible to locate the antigen with nanoscale precision, and this makes it quite straightforward to change the antigen patterning and study the effect on antibody binding. For similar research applications, the limitations due to cost and stability of the DNA structures are also less important, as studies may be limited to less complex media (i.e., buffer solutions).

## Conclusions

DNA nanotechnology allows the rational design of DNA-based switches, devices, and nanostructures that can be programmed to respond to a wide range of environmental and molecular inputs. Different signaling moieties (i.e., fluorophore/quencher pair or electrochemical redox labels), anchoring tags (i.e., thiol groups for the attachment to an electrode surface), and molecules that act as recognition elements (i.e., antigens, peptides) can be conveniently conjugated to synthetic DNA sequences. This allows to introduce a number of responsive molecular components on DNA-based nanostructures and nanodevices that can thus be used to sense the presence of different targets. Among these, several classes of antigen-conjugated DNA systems for the detection and characterization of antibodies and for antibody-induced drug release have been reported to date. In this Perspective we have provided a general overview focusing on the most recent (last 5 years) and relevant examples in this field. The beauty of these systems is that they are extremely simple and versatile. DNA strands can be conjugated with antigens (both small molecules and entire proteins) quite easily, and the geometry of antibodies is such that the binding event with the antigen can be conveniently predicted. This makes the optimization and characterization of new sensing strategies often a straightforward process, and usually few iterations of design/synthesis/test are needed to achieve good results in terms of signal-to-noise and specificity. The versatility is also related to the signaling output. DNA strands can be conjugated to both fluorescence and electrochemical tags, and thus the same sensing scheme can be easily adapted to two different methods. With electrochemical detection, it is possible to use disposable sensors and portable and low-cost instruments that are very well suited for point-of-care applications. With fluorescence detection, it is possible to use well-plate readers that allow a large number of samples to be processed at the same time. Fluorescence detection can also be adapted to portable instrumentation (such as with smartphones), but to the best of our knowledge this type of approach has not been yet demonstrated with DNA-based sensors for antibody detection. Moreover, similar portable fluorescent instrumentation usually suffers from lower sensitivity compared to bench-type plate readers. Another advantage is the low cost of the reagents needed for these systems. This is especially true for all the DNA-based switches and colocalization-based approaches where only antigen-conjugated and signaling tag-conjugated DNA strands are used. The modification of DNA strands can be done in house using, for example, standard click chemistry reactions. Alternatively, it is possible to purchase the DNA strand already conjugated to the molecule of interest. As an example, 150 μg (approximately 20–30 nanomoles) of a 20-nt synthetic DNA strand conjugated to either a fluorescent or an electrochemical tag and HPLC-purified can be purchased for about 50–100 euros. For antigen-conjugated strands, a difference should be made. When a small molecule (such as Dig or DNP) is used as an antigen, the cost of the antigen-conjugated DNA strand is similarly low (i.e., 150 μg cost about 100 euros). If the antigen needed is a short peptide, instead, it is preferrable to conjugate it to a PNA strand which is more suitable for conjugation to a peptide. In this case the cost could be slightly higher (150 μg cost about 300 euros). When whole proteins are used as antigens, the conjugation step can be done using, for example, NH_2_-modified DNA strands but a purification step (using ion-exchange chromatography) is required, and this can reduce the final yield and ultimately increase the total cost. It should also be noted that in all the systems described in this Perspective the modified DNA strands are used in small volumes (usually 10–50 μL) at nanomolar concentrations. For this reason, 150 μg (or 20–30 nanomoles) of the modified DNA strands is usually enough for hundreds of tests. Obviously, the above-reported costs are those required for R&D optimization, and in the case of commercialization of a sensor these will be further reduced due to mass production.

Before these platforms can become a commercial reality, some problems and limitations should, however, be overcome. The most important and crucial limitation is related to the sensitivity of these systems. The majority of the DNA-based systems for antibody detection that we described here, regardless of the sensing mechanism, does not involve any signal amplification step (either chemical or enzymatic). This ultimately means that the detection limit for these systems cannot go below nanomolar (or high picomolar) concentration range (fixed by the intrinsic instrumental sensitivity for both fluorescence and electrochemical detection modes). To achieve lower detection limits would require an amplification step using, for example, an enzymatic reaction (similar to that employed in ELISA systems). This would make the detection scheme more complicated (as would add reaction and washing steps) but appears inevitable if pM/fM detection limits need to be reached.

Finally, we note that in addition to sensing, antigen-conjugated DNA strands may also find other applications. Here, for example, we gave an overview of some of the possible applications (i.e., characterization of antibody/antigen interaction, antibody-induced chemical reactions, and drug release) that may eventually open the door to new avenues for targeted therapy, diagnostics, and therapeutics.
